# rSJYB1 inhibits collagen type I protein expression in hepatic stellate cells via down‐regulating activity of collagen α1 (I) promoter

**DOI:** 10.1111/jcmm.14271

**Published:** 2019-03-20

**Authors:** Liuting Chen, Zhaodong Ji, Lian Duan, Dandan Zhu, Jinling Chen, Xiaolei Sun, Yang Yu, Yinong Duan

**Affiliations:** ^1^ Department of Pathogen Biology, School of Medicine Nantong University Nantong Jiangsu People's Republic of China; ^2^ Cancer Institute Fudan University Shanghai Cancer Center Shanghai People's Republic of China; ^3^ Department of Oncology, Shanghai Medical College Fudan University Shanghai People's Republic of China; ^4^ Department of Medical Informatics, School of Medicine Nantong University Nantong Jiangsu People's Republic of China

**Keywords:** collagen type I, hepatic stellate cell, liver fibrosis, SJYB1

## Abstract

YB1 is a negative regulator in liver fibrosis. We wondered whether SJYB1, a homologous protein of YB1 from *Schistosoma japonicum*, has an effect on liver fibrosis in vitro. Recombinant SJYB1 (rSJYB1) protein was expressed in a bacterial system and purified by Ni‐NTA His·Bind Resin. A human hepatic stellate cell line, the LX‐2 cell line, was cultured and treated with rSJYB1. The role of rSJYB1 on LX‐2 cells was then analysed by Western blot and luciferase assay. We succeeded in expressing and purifying SJYB1 in a bacterial system and the purified rSJYB1 could be recognized by *S japonicum*‐infected rabbit sera. Western bolt analysis showed that rSJYB1 inhibited the expression of collagen type I, but had little effect on α‐smooth muscle actin (α‐SMA). Further analysis revealed that rSJYB1 inhibited the activity of collagen α1 (I) (COL1A1) promoter and functioned at −1592/−1176 region of COL1A1 promoter. Our data demonstrate that rSJYB1‐mediated anti‐fibrotic activity involves inhibiting the activity of COL1A1 promoter and subsequently suppressing the expression of collagen type I in hepatic stellate cells.

## 1 INTRODUCTION

Liver fibrosis is a pathological process characterized by accumulation of excess extracellular matrix (ECM) during sustained activation of wounding healing response. Deposition of ECM in the space of Disse leads to loss of the normal fenestrations, which provokes impairment of normal bidirectional metabolic exchange between portal venous flow and hepatocytes. It is widely accepted that activated hepatic stellate cells (HSCs) are the main producers of ECM, including collagen type I.^1^ Thus, inhibition of activated HSCs and reduction of abundant ECM are primary strategies to ameliorate liver fibrosis.

Y‐box‐binding protein 1 (YB1) is a member of the family of DNA‐ and RNA‐binding proteins with a conserved nucleic acid‐binding region called cold shock domain. In the past decade, numerous studies have identified YB1 as a negative regulator in liver fibrosis. Dooley et  al reported that YB1 exerts anti‐fibrotic action by inhibiting transforming growth factor‐β (TGF‐β) signalling, a major pathway to initiate HSC activation.^2^ Overexpression of YB1 can partially rescue tissue in a CCl_4_‐induced liver damage model^3^ and this beneficial effect is linked with the repressive effect of YB1 on collagen type I gene transcription.^4^ Furthermore, it is reported that a novel small compound Hsc025 ameliorates CCl_4_‐induced liver fibrosis in mice by promoting nuclear translocation of YB1 and subsequently inhibiting TGF‐β‐stimulated collagen gene expression.^5^

In this study, we expressed and purified a YB1 homologue, the recombinant SJYB1 protein (rSJYB1) from *Schistosoma japonicum*(*S japonicum*) in a bacterial system. Results from this study demonstrated that rSJYB1 inhibits collagen type I protein expression in HSCs via down‐regulating activity of collagen α1 (I) (COL1A1) promoter.

## 2 MATERIALS AND METHODS

### 2.1 Reagents

Rabbit pAbs against glyceraldehyde 3‐phosphate dehydrogenase (GAPDH) (Goodhere, Hangzhou, Zhejiang, China), mouse mAbs against His (TIANGEN, Beijing, China), collagen type I (Abcam, Cambridge, UK) or α‐smooth muscle actin (α‐SMA) (Santa Cruz Biotechnology, Dallas, TX, USA), horseradish peroxidase (HRP)‐conjugated anti‐rabbit IgG (Biosharp, Hefei, China) and HRP‐conjugated anti‐mouse IgG (Santa Cruz Biotechnology, Dallas, TX, USA) were purchased from the indicated companies.

### 2.2 Amplification of SJYB1

Based on the nucleotide sequence of SJYB1 (NCBI Accssion No. AF367371), two primers were designed to amplify SJYB1 from *S japonicum* egg cDNA (Table 1). PCR parameters were as follows: denaturation at 98°C for 10 seconds, primer annealing at 58°C for 5 seconds, primer extension at 72°C for 1 minute, cycled for 30 cycles, with a final extension at 72°C for 10 minutes before storing the sample at 4°C. The PCR product was sequenced and the sequence was characterized using nucleotide basic local alignment search tool (https://blast.ncbi.nlm.nih.gov/Blast.cgi) and named SJYB1.

**Table 1:** Primers used in this study

Primer	Sequence (5′→3′)	Purpose
SJYB1 F	CGGGATCCATGGCGGACGCAAGAGCAGC	pET28a‐SJYB1
SJYB1 R	CCCAAGCTTCTATGCATCGCGCATATCTGGC	pET28a‐SJYB1
COL1A1 F	CGAGCTCGGCAATGGAATCTTGGATG	pGL3‐COL1A1
COL1A1a F	CGAGCTCGGCCTTCTCTCAGCCCTAC	pGL3‐COL1A1a
COL1A1b F	CGAGCTCGGTTCTCTCTGGTTTGACTG	pGL3‐COL1A1b
COL1A1c F	CGAGCTCGGAAGAGAGCAGAGGGGC	pGL3‐COL1A1c
COL1A1d F	CGAGCTCGCCGGGCCAGGCAGCTCTG	pGL3‐COL1A1d
COL1A1e F	CGAGCTCCTGGGGCACGGGCGGCC	pGL3‐COL1A1e
COL1A1 R	CCGCTCGAGCGGAGGTCCACAAAGCTG	Reporters^a^

F, Forward; R, Reverse.

a Reporters: pGL3‐COL1A1, pGL3‐COL1A1a, pGL3‐COL1A1b, pGL3‐COL1A1c, pGL3‐COL1A1d, pGL3‐COL1A1e.

### 2.3 Construction of SJYB1 prokaryotic expression vector

PCR products of SJYB1 were digested with BamHI and HindIII enzymes and ligated to BamHI‐ and HindIII‐digested expression vector pET28a (+). Insert DNA was sequenced to ensure authenticity of the clone nucleotide sequence.

### 2.4 Expression and purification of rSJYB1

The empty pET28a (+) vector or the recombinant construct of SJYB1 (pET28a‐SJYB1) was transformed into *Escherichia coli* BL21 (DE3). For expression, the bacteria were grown in Luria‐Bertani (LB) medium supplemented with kanamycin (100 μg/mL) at 37°C, 200 rpm on a shaking incubator with a rotational radius of 10 cm. The expression of rSJYB1 was induced by adding 0.005 mmol/L isopropyl β‐d‐thiogalactopyranoside (IPTG) when the culture OD600 had reached 0.4. After 12 hours of induction, the total bacteria cells were harvested by centrifugation at 8600 *g* for 10 minutes. Then, the pellet was resuspended in binding buffer and broken down by sonication. Followed by centrifugation at 8600 *g* for 10 minutes, the protein was purified from the supernatant by Ni‐NTA His·Bind Resin (Novagen, Madison, WI, USA) according to the manufacturer's instructions and dialysed against PBS, which was changed every 12 hours. After identified by Western bolt analysis, the endotoxin in rSJYB1 was removed using polymyxin B‐agarose beads (Sigma, Saint Louis, MO, USA) following the suggested protocol. The removal of endotoxins in the protein was verified using the ToxinSensor^TM^ chromogenic limulus amebocyte lysate endotoxin assay kit (GenScript, Nanjing, Jiangsu, China).

### 2.5 Infection sera

New Zealand white rabbits were percutaneously infected with 200 cercariae and the sera were collected 45 days pi. Animal welfare and experimental procedures were carried out in accordance with the Guide for the Care and Use of Laboratory Animals (Ministry of Science and Technology of China, 2006) and were approved by Animal Care Committee of Nantong University under license no. 20170403‐001.

### 2.6 Cell culture

The LX‐2 cells were purchased from the XiangYa Central Experiment Laboratory (China). Cells were cultured in DMEM (Gibco, Waltham, MA, USA), supplemented with 10% foetal bovine serum (Thermo, Waltham, MA, USA) at 37°C with 5% CO_2_ in a humidified incubator.

### 2.7 Western blot analysis

Cells were lysed in radio‐immunoprecipitation assay buffer with 1% phenylmethanesulfonyl fluoride (PMSF) (Biosharp) and phosphatase inhibitor complex III (1 mmol/L) (Sangon Biotech, Shanghai, China). Equal amounts of protein extracts were separated by 8% sodium dodecyl SDS‐PAGE and then transferred onto polyvinylidene difluoride (PVDF) membranes (Merck, Darmstadt, Germany). The membranes were blocked in 5% non‐fat milk for 2 hours at room temperature and incubated with the indicated primary antibodies at 4°C overnight. After being washed in TBS/Tween 20, the membranes were incubated with HRP‐conjugated secondary antibodies for 1 hour at room temperature. The protein bands were visualized with ECL regents (Millipore, Boston, MA, USA).

### 2.8 Construction of plasmids containing COL1A1 promoter sequence

Genomic DNA was extracted from LX‐2 cells according to instructions for QIAamp® DNA Micro Kit (Qiagen, Hilden, Germany) and used as a template. For generating the COL1A1 promoter construct (pGL3‐COL1A1), a 1744 bp fragment containing the sequences from −1722 to +22 of human COL1A1 promoter was amplified by PCR from genomic DNA. The primers were designed according to the genomic sequence of human chromosome 17 (GenBank accession no. NC000017.11) for COL1A1 (Table 1). The PCR products were digested with SacI and XhoI and then subcloned into pGL3‐basic vector (Promega, Madison, WI, USA). To construct the COL1A1 promoter‐associated truncated plasmids, the indicated fragments were amplified by PCR from pGL3‐COL1A1 and PCR primers were designed as shown in Table 1. The PCR products were also digested with SacI and XhoI and then subcloned into pGL3‐basic vector.

### 2.9 Transfection and dual‐luciferase reporter assay

LX‐2 cells were cotransfected with the indicated plasmids of COL1A1 promoter (1 μg) and the pRL‐TK reporter plasmid (0.02 μg) using FuGENE (Promega) according to the manufacturer's instructions. After transfection for 24 hours, LX‐2 cells were treated with rSJYB1 or left untreated and cultured for another 72 hours. Then, the cells were harvested for luciferase activity analysis using a dual‐specific luciferase Reporter assay kit (Promega). pGL3‐basic Vector (Promega) that we used to construct the plasmids of COL1A1 promoter contained a modified coding region for firefly luciferase and the pRL‐TK reporter plasmid contained a modified coding region for Renilla luciferase. In dual‐luciferase reporter assay, the activities of firefly and Renilla luciferases are measured sequentially from a single sample. The activity of the pRL‐TK reporter plasmid provided an internal control that served as the baseline response. Normalizing the activity of the COL1A1 promoter to the activity of the internal control minimized experimental variability caused by differences in transfection efficiency.

## 3 RESULTS

### 3.1 Detection of anti‐SJYB1 antibody in *S japonicum*‐infected rabbit serum

**Figure 1:**
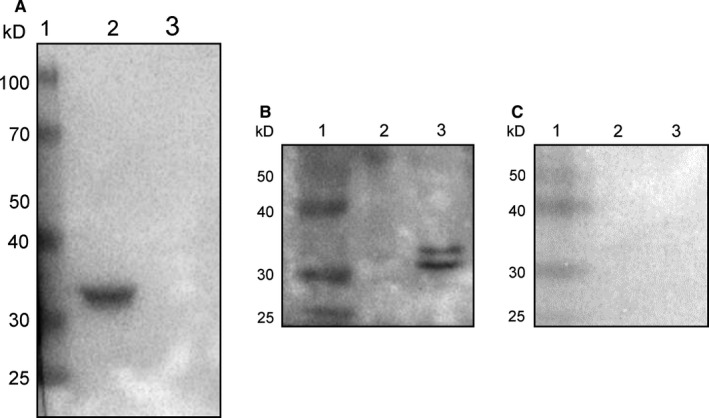
Detection of anti‐SJYB1 antibody in *Schistosoma japonicum*‐infected rabbit serum. A, Western blot analysis of purified recombinant SJYB1 (rSJYB1) using an anti‐His antibody. Lane 1: molecular weight marker; Lane 2: purified rSJYB1; Lane 3: control protein from empty pET28a (+) vector. B, Western blot of rSJYB1 probed with *S japonicum*‐infected rabbit serum. Lane 1: molecular weight marker; Lane 2: control protein from empty pET28a (+) vector; Lane 3: purified rSJYB1. C, Western blot of rSJYB1 probed with normal rabbit serum. Lane 1: molecular weight marker; Lane 2: control protein from empty pET28a (+) vector; Lane 3: purified rSJYB1. The sera were used at 1:1000 dilution

Recombinant SJYB1 was expressed in a bacterial system. The purity of the recombinant SJYB1 was assessed using SDS‐PAGE and Western blot analysis with an anti‐His antibody, which revealed a protein of approximately 36 kD (Figure 1A), consistent with the predicted molecular mass of the recombinant protein. Purified rSJYB1 was probed with sera collected from rabbits infected with *S japonicum*. Sera from uninfected rabbits and protein sample from empty pET28a (+) vector were used as negative controls. The results showed that rSJYB1 was recognized strongly by *S japonicum*‐infected rabbit sera (Figure 1B). In contrast, sera from uninfected rabbits could not recognize rSJYB1 (Figure 1C) and neither *S japonicum*‐infected rabbit sera nor uninfected rabbit sera could recognize protein sample from empty pET28a (+) vector (Figure 1B,C). These results indicated that native SJYB1 induces circulating antibodies in rabbits during *S japonicum* infection.

### 3.2 rSJYB1 inhibits collagen type I expression in LX‐2 cells

**Figure 2:**
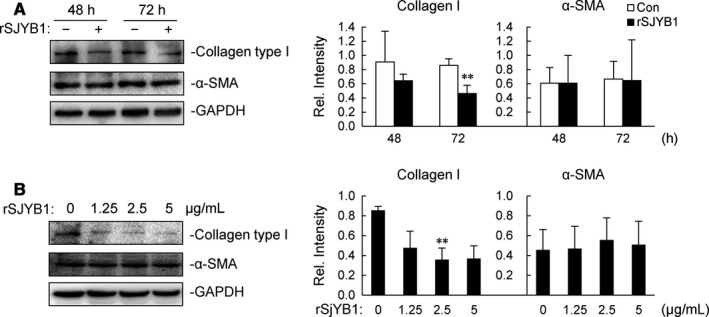
Recombinant SJYB1 (rSJYB1) inhibits collagen type I expression in LX‐2 cells. A, LX‐2 cells (1 × 10^6^) were left unstimulated or stimulated with 2.5 μg/mL rSJYB1 for the indicated times, and then, immunoblot analysis was performed with the indicated Abs. B, LX‐2 cells (1 × 10^6^) were left unstimulated or stimulated with several doses of rSJYB1, and then, immunoblot analysis was performed with the indicated Abs. The histograms show the relative intensities of the bands, which were quantitated by densitometry using Image Lab and normalized to GAPDH levels. Graphs show mean ± SD, *n* = 3. **P < 0.01

To determine the effect of rSJYB1 on liver fibrosis in vitro, we used a human HSC line, LX‐2 cells, to examine the protein expression of α‐SMA and collagen type I, the former being the marker of activated HSCs and the latter the major component of ECM. As shown in Figure 2A, LX‐2 cells were treated with 2.5 μg/mL rSJYB1 for different times, and the expression of collagen type I was noticeably reduced after 72 hours. Unexpectedly, rSJYB1 had little effect on the expression of α‐SMA after 48 or 72 hours. We also measured the levels of collagen type I and α‐SMA in LX‐2 cells exposed to rSJYB1 at various concentrations (0, 1.25, 2.5 and 5 μg/mL) for 72 hours. The data revealed that treatment with rSJYB1 at 2.5 μg/mL resulted in an obvious decrease in the expression of collagen type I (Figure 2B), whereas no dose of rSJYB1 had an effect on α‐SMA expression. These results suggested that rSJYB1 is specifically involved in regulation of collagen type I expression to exert anti‐fibrotic action in LX‐2 cells.

### 3.3 rSJYB1 inhibits COL1A1 promoter activity in LX‐2 cells

**Figure 3:**
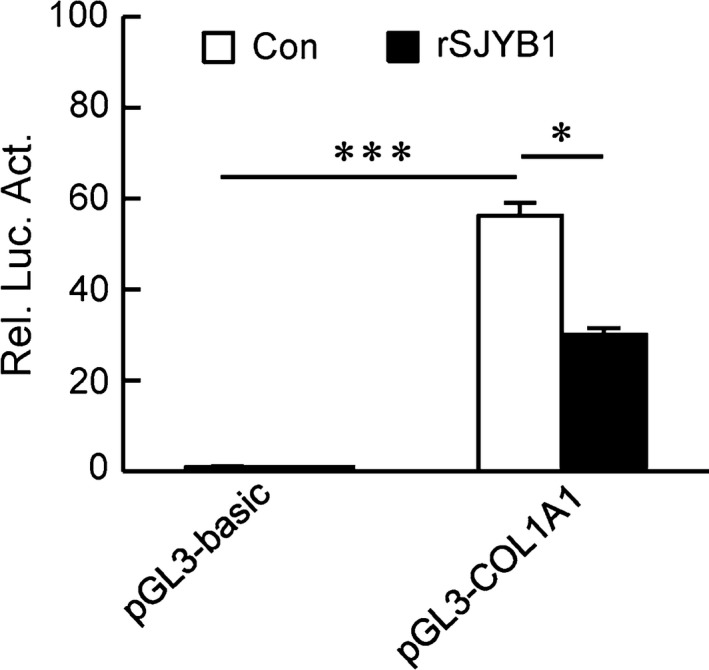
Recombinant SJYB1 (rSJYB1) inhibits collagen α1 (I) (COL1A1) promoter activity in LX‐2 cells. LX‐2 cells (1 × 10^5^) were transfected with the empty pGL3‐basic vector or pGL3‐COL1A1 reporter plasmid (1 μg) and pRL‐TK reporter plasmid (0.02 μg). Twenty‐four hours after transfection, cells were left unstimulated or stimulated with rSJYB1 (2.5 μg/mL) for 72 h before luciferase assays were performed. The Graph shows mean ± SD, *n* = 3. *P < 0.05, ***P < 0.001

Next, to examine whether the effect of rSJYB1 on collagen type I expression takes place at the transcriptional level, we constructed a luciferase reporter plasmid of COL1A1 promoter, pGL3‐COL1A1. In our reporter assays, pGL3‐COL1A1 had markedly higher activity compared to pGL3‐basic in LX‐2 cells (Figure 3). However, its activity was decreased after treatment with rSJYB1 for 72 hours (Figure 3). These results showed that rSJYB1 has a crucial role in the regulation of COL1A1 promoter activity.

### 3.4 rSJYB1 functions at −1592/−1176 region of COL1A1 promoter to inhibit its activity

**Figure 4:**
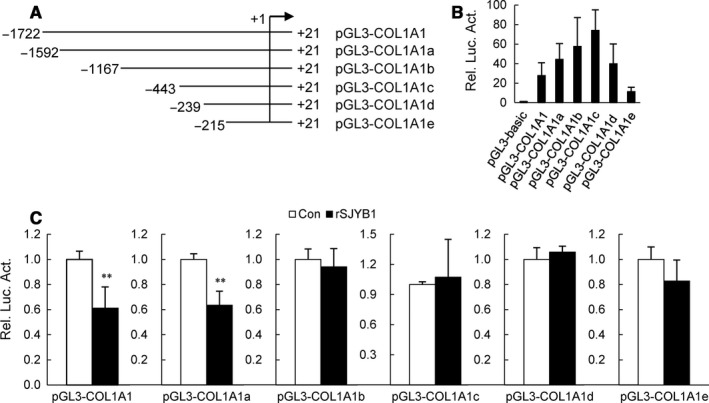
Recombinant SJYB1 (rSJYB1) functions at −1592/−1176 region of collagen α1 (I) (COL1A1) promoter to inhibit its activity. A, Diagram of the construction of COL1A1 promoter truncated fragments. B, LX‐2 cells (1 × 10^5^) were transfected with the empty pGL3‐basic vector, pGL3‐COL1A1, pGL3‐COL1A1a, pGL3‐COL1A1b, pGL3‐COL1A1c, pGL3‐COL1A1d or pGL3‐COL1A1e reporter plasmid (1 μg) and pRL‐TK reporter plasmid (0.02 μg). Twenty‐four hours after transfection, luciferase assays were performed. C, LX‐2 cells (1 × 10^5^) were transfected with the empty pGL3‐basic vector, pGL3‐COL1A1, pGL3‐COL1A1a, pGL3‐COL1A1b, pGL3‐COL1A1c, pGL3‐COL1A1d or pGL3‐COL1A1e reporter plasmid (1 μg) and pRL‐TK reporter plasmid (0.02 μg). Twenty‐four hours after transfection, cells were left unstimulated or stimulated with rSJYB1 (2.5 μg/mL) for 72 h before luciferase assays were performed. The graph shows mean ± SD, *n* = 3. **P < 0.01

In order to narrow down the activity region, we established five luciferase reporter plasmids of truncated fragments of COL1A1 promoter, named pGL3‐COL1A1a, pGL3‐COL1A1b, pGL3‐COL1A1c, pGL3‐COL1A1d and pGL3‐COL1A1e (Figure 4A). The activities of these truncated fragments of COL1A1 promoter were shown in Figure 4B. Next, these truncated fragments of COL1A1 promoter as well as pGL3‐COL1A1 were transfected into LX‐2 cells, respectively, and then, transfected cells were treated with rSJYB1 for 72 hours. Results from reporter assays indicated that COL1A1 promoter activity was apparently decreased in rSJYB1‐treated LX‐2 cells transfected with pGL3‐COL1A1 and pGL3‐COL1A1a, while rSJYB1 had little effect on activities of other truncated fragments of COL1A1 promoter (Figure 4C). These results suggest that rSJYB1 functions at −1592/−1176 region of COL1A1 promoter to inhibit its activity.

## 4 DISCUSSION

The YB1 proteins were first recognized as transcriptional factors binding to the Y‐box element. However, it has since become apparent that the YB1 proteins are multifunctional and participate in translational regulation, DNA repair and pre‐mRNA splicing^6^ Extensive studies have shown that the YB1 proteins are related to proliferation and transformation of eukaryotic cells,^7^, ^8^ being also correlated with tumour aggressiveness and as a promising molecular target in the therapy of cancer and inflammation.^9^ A schistosome YB1 protein SMYB1 was earlier identified by Franco et  al who isolated from an *Schistosoma mansoni* (*S mansoni*) adult worm library using the expressed sequence tag strategy.^10^ SMYB1 has been verified the ability to bind DNA and RNA like other Y‐box proteins and may act in the process of RNA metabolism.^11^, ^12^ Furthermore, mice immunized with the recombinant SMYB1 (rSMYB1) were detected the high levels of specific anti‐rSMYB1 antibodies in their sera.^13^ SJYB1 reported in our study shares 83.33% identity in its nucleotide sequence and 94.47% in its protein sequence with SMYB1 (data not shown). Recombinant SJYB1 was strongly recognized by *S japonicum*‐infected rabbit sera, suggesting that native SJYB1 can induce circulating antibodies in rabbits during *S japonicum* infection. Therefore, these results indicated that recombinant and native YB1 from schistosomes both have high immunogenicity.

Infection with *S japonicum* can lead to hepatic schistosomiasis, and the main pathologic lesions of hepatic schistosomiasis are granuloma formation and liver fibrosis around schistosome eggs.^14^ Schistosome eggs are an important source of antigens, which result in hepatic schistosomiasis.^15^ Nevertheless, Anthony et  al reported that schistosome eggs inhibit HSC fibrogenesis in vitro, with down‐regulation of α‐SMA and collagen type I^16^, ^17^ Our previous studies also found that soluble egg antigen (SEA), secreted by schistosome eggs, is engaged in anti‐fibrotic activity.^18^, ^19^, ^20^, ^21^ Furthermore, it is demonstrated that P40, a major component of SEA, limits HSC activation via inhibiting TGF‐β signalling to modulate liver fibrosis in vitro.^22^ However, the results of the current study suggested that the effect of rSJYB1 on HSCs differs from SEA and P40. Recombinant SJYB1 inhibited the expression of collagen type I, but not α‐SMA in LX‐2 cells, suggesting that rSJYB1 specifically regulates synthesis of collagen type I rather than HSC activation. Unexpectedly, another study on mice infected with *S mansoni* has revealed that infected mice immunized with rSMYB1 exhibited reductions in the number of adult worm and eggs/granuloma retained in the liver, but no significant decrease was detected in the area of fibrosis.^13^ It is possible that the roles of YB1 from schistosomes in vitro are not entirely consistent with that in vivo. In addition, whether the minor differences in protein sequences lead to the function difference between SJYB1 and SMYB1 has not been determined.

Norman et  al^4^ reported that human YB1 suppressed COL1A1 transcription via binding to an evolutionarily conserved regulatory element (−88/−48) in the proximal promoter. In our study, rSJYB1 failed to inhibit proximal promoter activity of COL1A1. Instead, rSJYB1 inhibited the activity of COL1A1 promoter −1592/+21, which might function at −1592/−1167 region of COL1A1 promoter. Interestingly, truncation analysis showed that the activity of COL1A1 promoter −443/+21 was higher than that of other COL1A1 promoters, which is consistent with the result reported by previous studies that the −331 bp portion of the 5′ promoter segment had the highest activity of human COL1A1 promoter,^23^ indicating the presence of negative regulatory elements between −1722 and −443 bp in COL1A1 promoter. Given the function of rSJYB1 in COL1A1 promoter activity, the question arises how its function is regulated. We used bioinformatics tools to predict the putative nuclear localization sequence in rSJYB1. Unfortunately, there was no putative nuclear localization sequence in rSJYB1 (data not shown), which suggested that rSJYB1 is less likely to work in the nucleus. Furthermore, it is demonstrated that SMYB1 could not bind to promoters of the *S mansoni* genes in vivo indicating that SMYB1 may not act directly as a transcription factor.^11^Therefore, it is possible that rSJYB1 regulates COL1A1 promoter activity indirectly, which may be via relevant transcription factors or signalling pathways, just as P40 regulates p27 promoter activity via transcription factor E2F1.^24^ Further studies will be performed to elucidate the molecular mechanism.

In conclusion, our data suggest that rSJYB1‐mediated anti‐fibrotic activity involves inhibiting the activity of COL1A1 promoter and subsequently suppressing the expression of collagen type I in HSCs.

## CONFLICT OF INTEREST

The authors declare that they have no conflict interests.
